# Immunoglobulin G1 and immunoglobulin G4 antibodies in multiple sclerosis patients treated with IFNβ interact with the endogenous cytokine and activate complement^☆^

**DOI:** 10.1016/j.clim.2013.05.008

**Published:** 2013-08

**Authors:** Swaminathan Sethu, Karthik Govindappa, Paul Quinn, Meenu Wadhwa, Richard Stebbings, Mike Boggild, Dean Naisbitt, Ian Kimber, Munir Pirmohamed, Kevin Park, Jean Sathish

**Affiliations:** a MRC Centre for Drug Safety Science and Institute of Translational Medicine, Department of Molecular and Clinical Pharmacology, University of Liverpool, Sherrington Buildings, Liverpool L69 3GE, UK; b National Institute for Biologics Standards and Control, Hertfordshire, EN6 3QG, UK; c The Walton Centre NHS Foundation Trust, Lower Lane, Liverpool, L9 7LJ, UK; d The Townsville Hospital, North Queensland, 4814, Australia; e Faculty of Life Sciences, University of Manchester, Manchester M13 9PT, UK; f The Wolfson Centre for Personalized Medicine, University of Liverpool, Block A Waterhouse Buildings, Liverpool, L69 3GL, UK

**Keywords:** Interferon beta;, Relapsing-remitting multiple sclerosis;, Immunogenicity;, Anti-drug antibody;, Neutralising antibody;, Complement

## Abstract

A subset of patients with relapsing-remitting multiple sclerosis (RRMS) on therapy with interferon beta (IFNβ) develop neutralising anti-drug antibodies (ADA) resulting in reduced, or loss of, therapeutic efficacy. The aims were to characterise the relative contributions of anti-IFNβ antibody isotypes to drug neutralising activity, ability of these antibodies to cross-react with endogenous IFNβ, to form immune complexes and activate complement. IFNβ-specific ADA were measured in plasma from RRMS patients treated with IFNβ1a (Rebif^®^). Neutralisation of endogenous and therapeutic IFNβ by ADA was determined by IFNβ bioassay. IFNβ-ADA profile was predominantly comprised of IgG1 and IgG4 antibody isotypes. The contribution of IgG4-ADA towards neutralising activity was found to be minimal. Neutralising IFNβ-ADA blocks endogenous IFNβ activity. ADA interaction with therapeutic IFNβ results in immune complex formation and complement activation. In summary, IgG1 and IgG4 IFNβ-ADA have the ability to neutralise therapeutic and endogenous protein and to activate complement.

## Highlights

• IgG4 and IgG1 contributes to IFNβ-ADA profile
• Neutralising IFNβ-ADA cross reacts and blocks endogenous IFNβ activity.
• ADA-IFNβ results in IC formation and complement activation

## 1 Introduction

Multiple sclerosis (MS) is a chronic inflammatory demyelinating disease of the central nervous system and the most common form of MS at onset is relapsing-remitting MS (RRMS) [1]. Clinical trials have demonstrated the efficacy of recombinant interferon beta (IFNβ1a and IFNβ1b), in reducing relapse rate and disease progression in RRMS patients [2,3]. A major limitation with continuous IFNβ treatment is the development of anti-drug antibodies (ADA) [4–6]. ADA can be non-neutralising (N-NAbs) or neutralising (NAbs) depending on their ability to block bioactivity of therapeutic IFNβ by interacting at sites that are crucial for drug-target interaction. In general, ADA against biologics can result in loss of bioactivity, altered pharmacokinetics, neutralisation of endogenous counterparts, infusion reactions, hypersensitivity and anaphylaxis [7–10].

ADA against therapeutic IFNβ are reported to be predominant of the immunoglobulin G (IgG) class. Reports on the distribution of IgG subclasses (IgG1-4) of IFNβ-specific NAbs in MS patients on IFNβ therapy are limited [11,12]. Type I interferons, including IFNβ, are crucial in the regulation of host immune responses. The potential for neutralisation of endogenous IFNβ by IFNβ1a-specific ADA (IFNβ-ADA) is unclear. Interaction of ADA with the therapeutic can result in the formation of immune complex (IC) that can potentially activate complement. Complement factors are a group of plasma proteins that play a pivotal role in both innate and adaptive immunity [13]. There is considerable evidence for the influence of activated complement in antigen processing and presentation [14,15]. Therefore, increase in complement activation can enhance antigen processing and presentation which may contribute to the progression of immunogenic responses to biologics.

In this study, our aim was to determine the immunoglobulin types/subtypes of IFNβ-ADA in samples from patients treated with IFNβ and their relative contribution in neutralising IFNβ bioactivity, the potential of NAb to cross react with endogenous IFNβ and the ability of ADA-IFNβ immune complexes to activate the complement cascade.

## 2 Materials and methods

### 2.1 Chemicals and reagents

All chemicals and reagents used in the study, unless specified otherwise, were obtained from Sigma-Aldrich, UK.. anti-human MxA antibody (Santa Cruz Biotechnologies, USA); anti-rabbit and anti-mouse HRP-conjugated secondary antibody (Sigma Aldrich, UK); mouse anti-human β-actin (Abcam, UK); THP-1 — human acute monocytic leukaemia cell line (American Type Culture Collection, #TIB-202™); IFNβ1a formulation — Rebif^®^ (EMD Serono, Inc. and Pfizer, Inc.).

### 2.2 Subject details

Ten healthy donors and nineteen RRMS patients with history of IFNβ therapy as detailed in Table 1 were chosen for this study from The Walton Centre NHS Foundation Trust, UK. Approval for the study was acquired from the Liverpool local research ethics committee and informed written consent was obtained from each donor. Plasma and serum fractions obtained from peripheral venous blood samples were stored in aliquots at − 80 °C until further use. Fresh aliquots of serum samples were used for each experiment to ensure that samples did not undergo more than one freeze–thaw cycle.

### 2.3 Detection and quantification of total and IFNβ-specific immunoglobulins

Total IgG and IgM in the plasma were quantified by sandwich type enzyme-linked immunosorbent assay (ELISA) using human IgG and IgM ELISA quantitation kits (Bethyl Laboratories, Inc. USA), and total IgG subclass levels in the plasma were quantified by ELISA using human IgG subclass profile kit (Invitrogen — Life Technologies, UK) according to manufacturer's instructions. IFNβ-specific immunoglobulins (IgG and IgM) and IgG subclass were quantified using ELISA according to manufacturer's instructions (IgG/IgM — Bethyl Laboratories, Inc., USA; IgG subclass — Cygnus Technologies, Inc., USA) with the following modifications. IFNβ1a (Rebif^®^, 1.5 μg/ml) was coated and the plasma dilutions used were 1:20 for IgG/IgG subclass and 1:2 for IgM detection. The absolute concentrations for total and IFNβ-specific immunoglobulins types were calculated based on the standards provided in the kit, except for IFNβ-specific IgG subclass where the analysis was based on optical density values.

### 2.4 Neutralisation assay — IFNβ induced MxA bioassay

Myxovirus resistance protein A (MxA) expression is a well-known biomarker for IFNβ bioactivity [16]. The neutralising potential of IFNβ-ADA was determined by measuring IFNβ-induced (MxA) protein expression in THP-1 cells, using western blotting. Preliminary experiments in our laboratory established that IFNβ induced, in a dose-dependent manner, the expression of MxA protein in THP-1 cells (data not shown). Briefly, IFNβ1a (Rebif^®^) 100 U/ml was incubated with diluted patient plasma for 1 hour at 37 °C followed by its addition to THP-1 cells in culture and further incubated for 18 hours at 37 °C. The cells were subjected to detergent lysis, 20 μg of protein lysate separated using 10% SDS–PAGE and probed for MxA expression by western blotting using 1:500 rabbit anti-human MxA antibody (Santa Cruz Biotechnologies, USA) followed by 1:5000 HRP-conjugated anti-rabbit secondary antibody (Sigma Aldrich, UK), developed with enhanced chemiluminescence (ECL) and visualised by autoradiography. Intensities of immunoreactive bands were quantified by densitometry (TotalLabTL120software — Nonlinear Dynamics Ltd., Newcastle, UK).

### 2.5 Cross reactivity of ADA to endogenous IFNβ

The potential of ADA to cross react with endogenous IFNβ was assessed using lipopolysaccharide (LPS) — 1 μg/ml and polyinosinic–polycytidylic acid [poly(I:C)] — 10 μg/ml triggered IFNβ secretion by THP-1 cells and subsequent MxA expression due to the autocrine effect of the secreted IFNβ on the THP-1 cells (Fig. 4a). Cross reactivity of ADA was assessed by co-incubating diluted ADA positive patient plasma with LPS and poly(I:C) in THP-1 cells followed by culture at 37 °C for 24 hours. After 24 hours, MxA levels were quantified by western blotting as described earlier.

### 2.6 IgG4 depletion

Human IgG4 was depleted from plasma using human IgG4-specific affinity resin (Capture select, BAC BV, The Netherlands). Briefly, total plasma was incubated with the resin for 3 minutes at room temperature in a Mobicol “F” spin column (MoBiTec GmbH, Germany). Subsequently, plasma sample depleted of IgG4 was collected by brief centrifugation. Total IgG4 depleted (80–99%) plasma was subsequently used for the experiments.

### 2.7 Measurement of ADA-IFNβ immune complex

Formation of ADA-IFNβ immune complex *in vitro* was induced by incubating IFNβ1a (Rebif^®^) with patient serum for 1 hour at 37 °C followed by assessing the amount of IgG based complexes bound to C1q using IMTEC-CIC IgG ELISA kit (Imtec, Human Gesellschaft für Biochemica und Diagnostica mbH, Germany) according to manufacturer's instructions.

### 2.8 Measurement of immune complex induced C3a

ADA-IFNβ immune complexes were induced as described above and activated complement factor, C3a, in these samples was measured by using C3a ELISA kit (Hycult Biotech, Netherlands) according to manufacturer's instructions.

### 2.9 Statistical analysis

Data were analysed with appropriate statistical tests (unpaired/paired *t*-test and ANOVA) as indicated in the figure legends using GraphPad Prism v.5.

## 3 Results

### 3.1 IFNβ1a-specific ADA are predominantly of the IgG1 and IgG4 subclass

**Figure 1:**
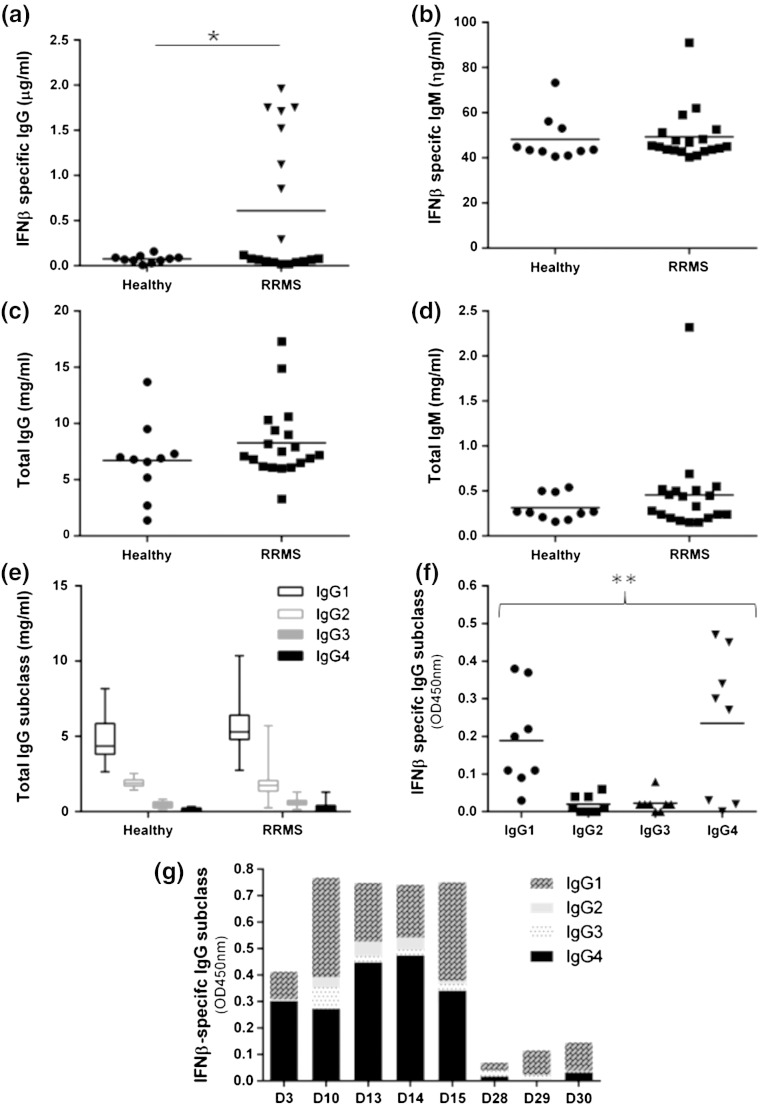
Immunoglobulin types/subtypes of therapeutic IFNβ-ADA. Total and IFNβ-specific IgG (a and c) and IgM (b and d) in the plasma of healthy donors (healthy) and RRMS patients (RRMS) were determined by ELISA. Data points ▼ in graph (a) are categorised as IFNβ-specific IgG based ADA positive samples. (e) Total IgG subclass in healthy and RRMS plasma samples. (f) IFNβ-specific IgG subclass levels in 8 ADA positive samples detected. (g) Proportion of IFNβ-specific IgG subclass in each ADA positive plasma sample. Statistical analyses include *t*-test and ANOVA. ^⁎^*p* = 0.0352 (*t*-test unpaired); ^⁎⁎^*p* = 0.0045 (ANOVA); healthy — *n* = 10; RRMS — *n* = 19. The data point in each graph is a mean of triplicate measurements and is a representative of three independent experiments.

Eight out of nineteen RRMS patients had detectable levels of IFNβ1a-specific IgG (Fig. 1a). The samples were categorised as ADA positive when the IFNβ1a-specific IgG concentrations were above a threshold value of the mean and two standard deviations of the value in healthy control plasma. The concentrations of ADA ranged between 0.3 μg/ml and 2.0 μg/ml and were of IgG type and not IgM (Figs. 1a and b). There were no significant differences in the levels of total IgG and IgM between the healthy controls and RRMS samples (Figs. 1c and d). Immunoglobulin G subclass typing revealed that levels of IFNβ1a-specific IgG1 and IgG4 in the plasma of 8 ADA positive samples were significantly higher compared to IgG2 and IgG3 (Fig. 1f). Distribution of IFNβ1a-specific IgG subclasses for each of the samples indicated that both IgG1 and IgG4 at varying levels predominantly contributed to the ADA followed by IgG3 and IgG2 (IgG3 > IgG2, Fig. 1g). The ADA profile in the ADA-positive donor (D29) was found to be primarily made up of IFNβ1-specific IgG1 and IgG3 with IFNβ1-specific IgG4 being undetectable (Fig. 1g). There were no significant differences in the total IgG subclass levels between healthy and RRMS samples (Fig. 1e).

### 3.2 Neutralising potential and cross reactivity of IFNβ-ADA

**Figure 2:**
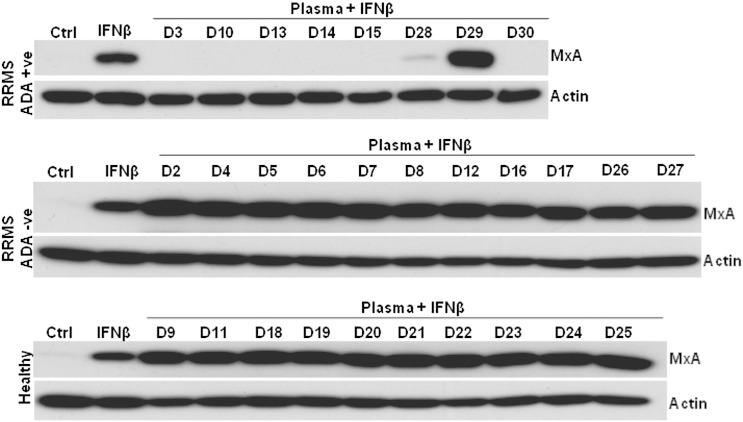
Neutralisation potential of therapeutic IFNβ-ADA. Immunoblot shows IFNβ-induced MxA protein levels in THP-1 cells. Therapeutic IFNβ (100U Rebif) pre-incubated with 1:10 dilution of plasma samples of healthy and RRMS with (ADA + ve) and without ADA (ADA − ve) for 1 hour at 37 °C was added to THP-1 cells for 18 hours. Proteins from whole cell lysates of these cells were separated by SDS–PAGE and western immunoblotting performed. Membranes were probed for human MxA and β-actin. “Ctrl” indicates cell lysates prepared from cells without any treatment and “IFNβ” indicates lysates from cells treated with 100U Rebif only. D2–D30 are lysate samples from cells incubated with the plasma and IFNβ mixture. The images are representative of three independent experiments.

We used IFNβ-induced intracellular MxA expression in THP-1 cells to assess the neutralisation potential of the IFNβ-specific ADA. Our data indicate that 7 out of 8 ADA-positive samples had the potential to neutralise IFNβ1a (Rebif^®^) induced MxA (Fig. 2). Sample D29 did not neutralise the IFNβ1a indicating that the ADA were of the non-neutralising type. As expected, none of the samples from ADA negative and healthy donors neutralised IFNβ-induced MxA (Fig. 2). Our results were consistent with the data obtained (data not shown) from other neutralisation assays carried out independently at National Institute for Biological Standards and Control (NIBSC), UK.

**Figure 3:**
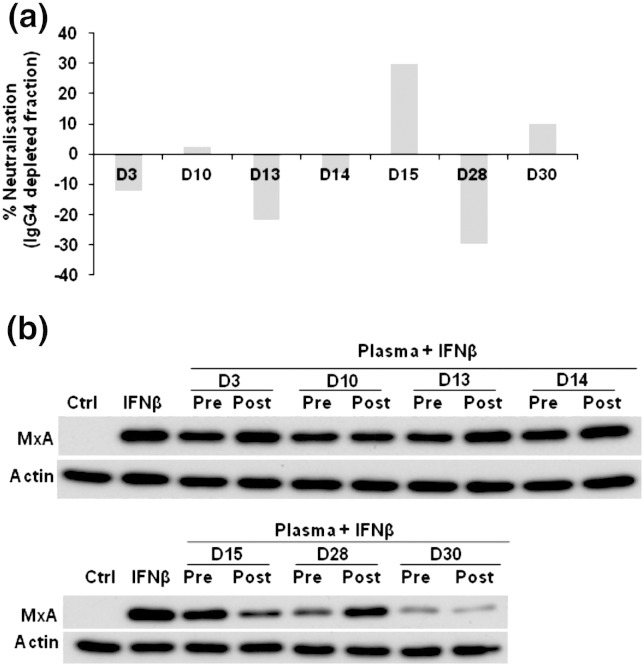
Neutralisation potential of IgG4 based therapeutic IFNβ-ADA. (a) “Neutralisation (%)” is the percentage based obtained on densitometry analysis immunoblot as shown in Fig. 3b assessing IFNβ-induced MxA neutralisation by RRMS ADA + ve plasma samples with and without IgG4 relative to “IFNβ” only category. The percentage indicates the difference in the neutralisation potential of IgG4 depleted plasma compared to its respective non-depleted fraction. (b) Immunoblot exhibits variable differences in the potency of ADA positive plasma samples with and without IFNβ-specific IgG4 to neutralise IFNβ-induced MxA protein levels in THP-1 cells. Therapeutic IFNβ (100U Rebif) pre-incubated with diluted of plasma from 8 RRMS ADA + ve samples (1:1000 — D3, D10, D13, D14, D15 and 1:10 — D28, D30) with (Pre) and without (Post) IgG4 for 1 hour at 37 °C was added to THP-1 cells for 18 hours. Proteins from whole cell lysates of these cells for each category were electrophoretically (SDS–PAGE) separated and probed for human MxA. β-Actin probed for all the samples served as loading controls. “Ctrl” indicates cell lysates prepared from cells without any treatment and “IFNβ” indicate lysates from cells treated with 100U Rebif only. The images are representatives of three independent experiments.

While earlier association studies have implicated IgG4 ADA as having greater neutralisation potential compared to other IgG subclasses, this has not been directly tested. We depleted IgG4 from ADA-positive plasma (samples D3, D10, D13, D14, D15, D28 and D30) and compared IFNβ neutralisation between total plasma and IgG4 depleted plasma using MxA assay (Fig. 3). Based on the results, depleting IgG4 in D3, D13, D14 and D28 contributed to a partial (12–30%) reduction in its neutralisation potential. The IgG4 depleted samples of D10, D15 and D30 exhibited an increase (2–30%) in neutralisation. Our observations from this study indicate that IgG4 may partly or not contribute to the neutralisation activity of neutralising IFNβ-specific ADA and that this effect can vary between patients.

**Figure 4:**
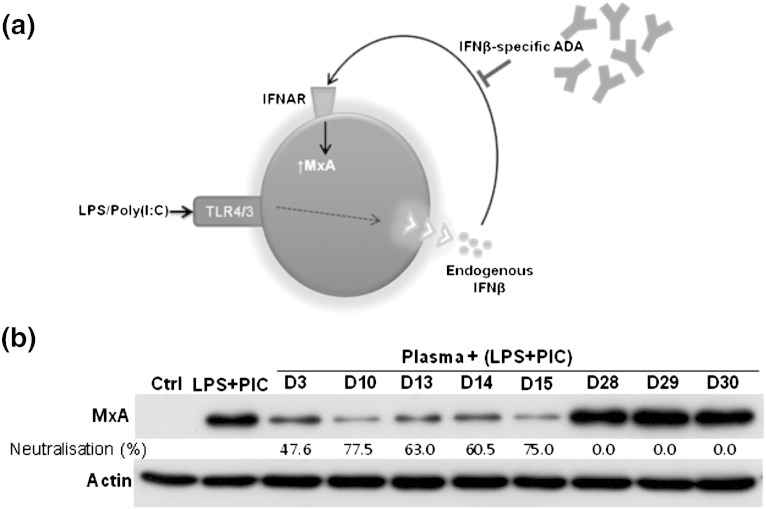
IFNβ-specific ADA cross reacts and neutralises endogenous IFNβ-induced bioactivity. (a) Bioactivity of endogenous IFNβ was assessed by triggering IFNβ induced MxA by TLR4/3 (LPS — 1 μg/ml)/(poly(I:C) — 10 μg/ml) activation. Cross reactivity of ADA to endogenous IFNβ was studied by investigating whether ADA were able to block or inhibit the induction of MxA in this system. (b) Immunoblot image shows MxA protein levels induced by LPS and poly(I:C) (LPS + PIC) stimulation for 24 hours in THP-1 cells. Induction of MxA is as a result of endogenous Type 1 interferons, including IFNβ triggered by LPS + PIC. The immunoblot also indicates the changes in the level of induced MxA when the cells were co-incubated with 1:10 dilution of plasma samples from 8 RRMS ADA + ve (D3, D10, D13, D14, D15, D28, D29 and D30) individuals along with LPS + PIC. Proteins from whole cell lysates for each category were electrophoretically (SDS–PAGE) separated and probed for human MxA. β-Actin probed for all the samples served as loading controls. “Ctrl” indicates cell lysates prepared from cells without any treatment and “LPS + PIC” indicates lysates from cells treated with LPS + PIC only. “Neutralisation (%)” is the percentage (densitometric analysis) of LPS + PIC induced MxA neutralised by RRMS ADA + ve plasma samples relative to “LPS + PIC” only category. Immunoblot shown here is a representative of three independent experiments.

Eight ADA-positive samples were tested for their cross reactivity to endogenous IFNβ. Five (D3, D10, D13, D14 and D15) out of the eight samples neutralised TLR4/3 (LPS/poly(I:C)) induced endogenous IFNβ at varying degrees (~ 47% to ~ 77%) as seen by the reduction in the levels of MxA compared to its positive control (Lane 2 — Fig. 4). Our results indicate that ADA generated in response to therapeutic IFNβ can neutralise endogenous IFNβ.

### 3.3 Complement activation by ADA-IFNβ immune complex

**Figure 5:**
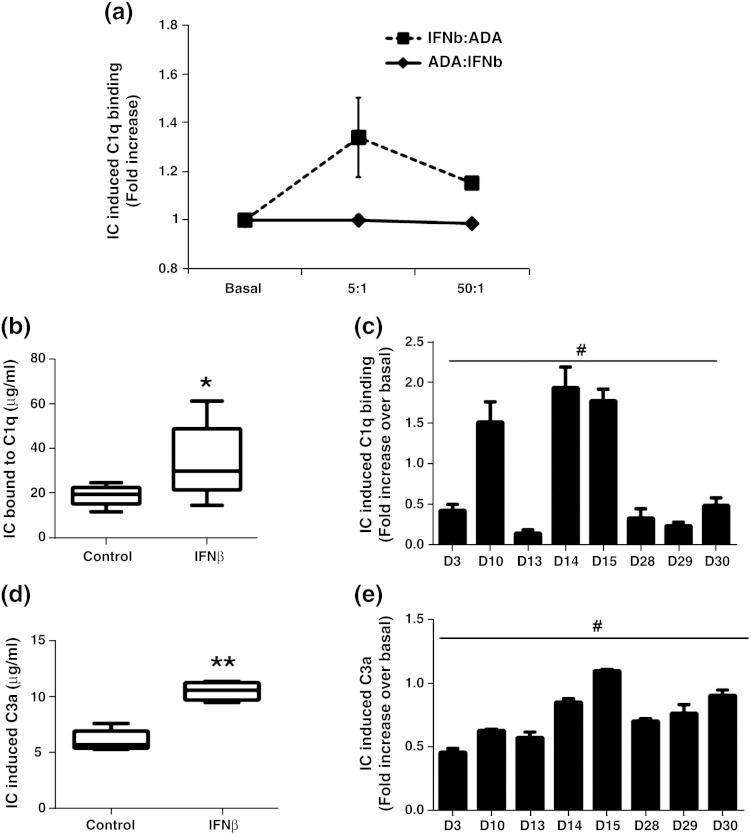
Therapeutic ADA-IFNβ immune complex activates complement system. (a) Ag[IFNβ]:Ab[ADA] ratio (IFNβ:ADA) at which immune complexes (IC) were formed by incubating IFNβ with serum containing ADA for 1 hour at 37 °C (*n* = 2). Graphs indicate the levels of ADA-IFNβ immune complexes bound to C1q (b and c) and subsequent changes in the level of C3a (d and e) in 8 RRMS ADA + ve (D3, D10, D13, D14, D15, D28, D29 and D30) serum samples. Statistical analyses include *t*-test and ANOVA. ^⁎^*p* = 0.0180 (*t*-test paired); ^⁎⁎^*p* < 0.0001 (*t*-test paired); ^#^*p* < 0.0001 (ANOVA); *n* = 8. The data point in each graph is a mean with standard deviation of triplicate measurements and is a representative of three independent experiments.

Immune complexes between a biologic and ADA have the potential to activate complement by first binding to complement component C1q, which then leads to the induction of activated complement. Optimal C1q binding of the ADA-IFNβ immune complex was observed at an antigen–antibody (IFNβ1a:ADA) ratio of 5:1 (Fig. 5a). A significant increase in the ADA-IFNβ immune complex formation was observed following IFNβ1a incubation in the ADA-positive serum samples (Fig. 5b). The degree of immune complex formation in individual samples was variable with values ranging from 0.2 to 2 fold increase from baseline (Fig. 5c). Elevated C1q binding was accompanied by a significant increase in the level of C3a in the serum samples incubated with IFNβ1a (Figs. 5d and e). These results demonstrate that IFNβ1a-specific ADA can form immune complexes with IFNβ1a and activate complement.

## 4 Discussion

IFNβ is one of the first line therapies used in the management of RRMS. The development of neutralising ADA has been reported with both IFNβ1a (2–39%) and IFNβ1b (38–42%) therapy [6]. In the current study, the immunoglobulin types of IFNβ-specific N-NAbs and NAbs detected were IgGs, with no detectable levels of IgM. It is however important to note that plasma samples were collected from individuals, most of whom had been on IFNβ therapy for many years. It is possible that IgM-ADA might have been part of the early/initiation phase (first few months after initiation of therapy) of an immunogenic response and would then have switched to IgG-ADA in the ADA positive samples. We have shown the presence of both IgG1 and IgG4-ADA contributing to the IFNβ-specific ADA profile. Earlier studies have shown a predominance of IgG4-ADA against IFNβ1a preparations [12] and, IgG2 and IgG4-ADA to IFNβ1b preparation [11]. These observations are similar to the immunogenic responses against glatiramer acetate (GA) treatment in RRMS which has shown predominance of IgG4-ADA [17,18]. The presence or an increase in the IgG4-ADA titre has been suggested to be associated with increased neutralisation potential of ADA [11]. In this study, we show that IgG4-ADA are less likely to be the major contributor to neutralisation potential of ADA despite the presence of increased titre of the IgG4 based IFNβ-ADA. Our data also suggest that IgG4-ADA are not always associated with neutralisation potential of IFN-specific ADA. This needs to be tested further with increased sample size and with purified IFNβ-specific IgG4. In addition, longitudinal studies are necessary to better understand the evolution of IgG subclass specific ADA and their neutralising capabilities.

Neutralising anti-drug antibodies against therapeutic thrombopoietin and epoetin have the ability to cross react with endogenous proteins and by compromising their bioactivity, they can lead to serious clinical consequences [8,19]. Neutralising ADA generated to either IFNβ1a or IFNβ1b can cross react and neutralise the activity of both these forms of IFNβ [20,21]. A recent report showed that nAbs triggered by both IFNβ1a and IFNβ1b therapy were able to neutralise fibroblast derived endogenous IFNβ [22,23]. Here, we have shown in an *in vitro* model that therapeutic IFNβ-specific neutralising ADA were able to neutralise endogenous IFNβ bioactivity in monocytic cells. The endogenous IFNβ activity was not completely neutralised compared to therapeutic IFNβ, suggesting that perhaps the level of endogenous IFNβ being produced was too high to be completely neutralised by the ADA. Neutralisation of endogenous IFNβ could have a potential impact on the integrity of the host immune system, could increase the susceptibility to viral infections and may affect the physiological role of endogenous IFNβ in various organ systems, including the CNS. This can be of particular significance when considering the fact that high titres of NAbs to IFNβ were found to last for many years after cessation of IFNβ therapy [5,24].

Antigen–antibody based immune complex triggers classical complement activation cascade with the release of activated complement factors including C3a and C5a. Our data show that interaction of ADA with therapeutic IFNβ resulted in immune complex formation and activated complement cascade following binding to C1q. Activated complements, such as C3a and C5a, have been implicated in enhancing antigen processing and presentation [14,15] and in the maintenance of tolerance [25]. Enhanced antigen uptake, processing and presentation may be major contributing factors in the initiation and progression of immunogenic response to biologics. Complement activation may actively facilitate this process and could potentially favour the development of ADA with increased neutralising potential. It is interesting to note that the incidence of ADA is higher with IFNβ preparations which are administered subcutaneously (e.g. Rebif) compared to the intramuscular route (e.g. Avonex) [26]. It is possible that immune complexes between the injected IFNβ and ADA can activate complement in the subcutaneous interstitium and gain access to skin resident APCs such as Langerhans cells and dermal dendritic cells. Such interactions between immune complexes, activated complement and skin APCs could enhance antigen processing and presentation of the therapeutic protein and enable the progression of immunogenicity. This could also be a mechanism by which low affinity and N-NAbs lead to the development of NAbs through immune complex formation and enhanced antigen processing/presentation. In the current study we observed sample D29 which had very limited or no neutralising potential but was able to form IC and activate complement in the presence of IFNβ. Based on this observation, we speculate that if a patient with N-NAbs continues to receive IFNβ therapy, immune complexes can be formed which through efficient antigen processing and presentation, and in combination with epitope spreading mechanisms, may eventually lead to the production of NAbs with consequences for continuation of therapy. Altered complement status and activated complement has been associated with MS [27,28]. Our observation of complement activation by IFNβ in ADA positive samples raises the possibility that repeated administration of IFNβ to ADA positive patients could cause additional alterations in the complement status in these patients. The clinical impact of such complement activation on MS disease progression is currently unknown and merits further investigation.

## 5 Conclusion

The development of neutralising anti-drug antibody development to biologics in general and interferon beta therapy in particular poses a significant clinical problem in terms of loss of efficacy and other adverse reactions. In the present study we have characterised the neutralising activity, IgG subclass profile, the potential for cross reactivity to endogenous interferon and complement activation of anti-drug antibodies to interferon beta in multiple sclerosis patients. Understanding the evolution of anti-drug antibody immune response and associated adverse immune outcomes can inform the development of better clinical approaches and regimens for biologics therapy.

## Conflict of interest statement

The authors declare that there are no conflicts of interest.

## Figures and Tables

**Table 1:** Details and history of IFNβ therapy.

Donor ID	Age	Sex	Group	IFNβ therapy status	Rebif (s.c.) dose (μg)	Duration (months^a^)	
						Therapy|Since WDL	
D9	24	F	Healthy	N.A.	N.A.	N.A.	N.A.
D11	42	M	Healthy	N.A.	N.A.	N.A.	N.A.
D18	47	F	Healthy	N.A.	N.A.	N.A.	N.A.
D19	30	F	Healthy	N.A.	N.A.	N.A.	N.A.
D20	43	F	Healthy	N.A.	N.A.	N.A.	N.A.
D21	33	F	Healthy	N.A.	N.A.	N.A.	N.A.
D22	43	M	Healthy	N.A.	N.A.	N.A.	N.A.
D23	30	F	Healthy	N.A.	N.A.	N.A.	N.A.
D24	27	M	Healthy	N.A.	N.A.	N.A.	N.A.
D25	32	F	Healthy	N.A.	N.A.	N.A.	N.A.
D4	47	M	RRMS	ON	22	48	N.A.
D5	57	M	RRMS	ON	44	32	N.A.
D6	30	F	RRMS	ON	44	> 120	N.A.
D8	43	M	RRMS	ON	22	> 120	N.A.
D12	32	F	RRMS	ON	44	141	N.A.
D16	56	F	RRMS	ON	22	> 120	N.A.
D17	37	F	RRMS	ON	44	96	N.A.
D26	50	F	RRMS	ON	22	60	N.A.
D27	45	F	RRMS	ON	22	120	N.A.
D2	55	F	RRMS	WD	22	43	5
D3	46	M	RRMS	WD	22	24	57
D7	54	M	RRMS	WD	22	36	44
D10	30	M	RRMS	WD	22	84	48
D13	49	M	RRMS	WD	22	15	34
D14	43	F	RRMS	WD	22	12	13
D15	43	M	RRMS	WD	22	72	53
D28	46	F	RRMS	WD	22	48	31
D29	53	F	RRMS	WD	22	36	57
D30	41	F	RRMS	WD	22	18	54

Abbreviations: RRMS — relapsing-remitting multiple sclerosis; Rebif — IFNβ1a; s.c. — subcutaneous; WD — withdrawn; WDL- withdrawal; ON — ongoing; N.A. — not applicable; F — female; M — male.

a Months calculated until the month of sample collection.
